# Association of FIB-4 and APRI with Contrast-Associated Acute Kidney Injury in Patients with Non-ST-Segment Elevation Myocardial Infarction Undergoing Coronary Angiography

**DOI:** 10.3390/jcm15218594

**Published:** 2026-11-04

**Authors:** Duygu Genç Albayrak, Duygu İnan, Osman Uzman, Ahmet Çağdaş Yumurtaş, Ufuk Yıldız, Burcu Aggül, Emir Renda, Funda Özlem Pamuk, Can Yücel Karabay, Ahmet İlker Tekkeşin

**Affiliations:** 1 Department of Cardiology, Başakşehir Çam and Sakura City Hospital, İstanbul 34480, Türkiye; 2 Department of Cardiology, Dr. Siyami Ersek Thoracic and Cardiovascular Surgery Training and Research Hospital, İstanbul 34668, Türkiye

**Keywords:** contrast-associated acute kidney injury, non-ST-segment elevation myocardial infarction, fibrosis-4 index, aspartate aminotransferase-to-platelet ratio index, risk stratification

## Abstract

**Background/Objectives**: In patients with non-ST-segment elevation myocardial infarction (NSTEMI), contrast-associated acute kidney injury (CA-AKI) is a clinically important complication of coronary angiography. Whether the Fibrosis-4 (FIB-4) index and aspartate aminotransferase-to-platelet ratio index (APRI) relate to CA-AKI in this setting is unclear. We compared the associations of both indices with CA-AKI. **Methods**: We retrospectively analyzed 771 patients with NSTEMI who underwent coronary angiography from May 2020 to May 2025. CA-AKI was identified from changes in serum creatinine 48–72 h after contrast administration. Both indices were derived from admission laboratory values. Associations were evaluated using separate multivariable logistic regression models. Discrimination was compared using the paired DeLong test, and the incremental performance of FIB-4 beyond the Mehran score was explored. **Results**: Overall, 97 patients (12.6%) developed CA-AKI. In the primary adjusted model, FIB-4 was associated with CA-AKI (odds ratio 1.19, 95% confidence interval 1.04–1.36; *p* = 0.013), but APRI was not. FIB-4 showed modest discrimination but a significantly higher area under the receiver operating characteristic curve (AUC) than APRI (0.614 versus 0.535; DeLong *p* < 0.001). Adding FIB-4 to the Mehran score improved model fit (likelihood-ratio *p* = 0.009), but the increase in AUC from 0.646 to 0.666 was not statistically significant (DeLong *p* = 0.100). Sensitivity analyses showed that the association varied with covariate adjustment. **Conclusions**: FIB-4 was associated with CA-AKI in the primary adjusted model and showed higher discrimination than APRI. These findings support further investigation of FIB-4 as a potential adjunct to renal risk assessment, although its modest discrimination and uncertain incremental clinical value require independent validation.

## 1. Introduction

Contrast-associated acute kidney injury (CA-AKI) is a clinically relevant complication observed after coronary angiography or percutaneous coronary intervention (PCI) involving iodinated contrast media. An increase in serum creatinine following a coronary procedure may complicate the subsequent hospital course and is associated with adverse outcomes [1,2,3,4]. In the large National Cardiovascular Data Registry (NCDR) Cath-PCI registry, acute kidney injury after percutaneous coronary intervention was associated with substantially higher in-hospital mortality, particularly when dialysis was required [5]. These observations make renal risk assessment an important part of the care of patients undergoing invasive coronary evaluation. However, the temporal relationship between contrast administration and kidney injury does not establish that contrast is the sole cause. The term CA-AKI acknowledges this distinction and allows for other contributors to renal deterioration during the same clinical episode [6].

In patients with non–ST-segment elevation myocardial infarction (NSTEMI), the assessment of renal risk takes place alongside decisions about the timing and extent of invasive treatment. Baseline kidney function, cardiac dysfunction, and haemodynamic compromise may influence the likelihood of AKI as well as the clinical circumstances in which angiography is performed [7,8,9]. Registry data have likewise identified severe chronic kidney disease and cardiogenic shock among the strongest correlates of AKI after percutaneous coronary intervention [5]. Thus, contrast exposure occurs within a broader clinical setting in which several potential renal insults may coexist. This complexity provides a reason to investigate admission characteristics associated with subsequent renal deterioration, while avoiding the assumption that any single laboratory measure identifies its underlying cause.

Clinical risk models combine several of these characteristics to estimate the probability of AKI after coronary procedures. The original Mehran score incorporates patient-related factors together with procedural information, including contrast volume [7]. It therefore provides a useful reference for risk assessment, although all of its components are not available before angiography. A systematic review of prediction models in acute coronary syndromes identified substantial methodological heterogeneity, a high risk of bias, and limited external validation [10]. Readily available laboratory indices are consequently of interest as potential additions to established assessment. Their convenience alone, however, does not establish clinical usefulness: a statistically significant association with CA-AKI must be distinguished from an improvement in prediction beyond information already captured by conventional risk factors.

The Fibrosis-4 (FIB-4) index and aspartate aminotransferase-to-platelet ratio index(APRI) were originally developed as non-invasive indices for hepatic fibrosis assessment [11,12]. Their potential relevance to CA-AKI does not imply that they directly measure renal fibrosis. Rather, these indices may capture clinical and laboratory characteristics associated with systemic inflammation, metabolic and vascular disease, and impaired renal reserve. FIB-4 has also been associated with cardiovascular outcomes in acute coronary syndromes [13,14]. These observations provide a rationale for examining its association with CA-AKI, while recognizing that its interpretation in an acute coronary setting may differ from its interpretation in chronic liver disease.

The two indices share aspartate aminotransferase (AST) and platelet count but differ in their remaining components. FIB-4 additionally incorporates age and alanine aminotransferase (ALT), whereas APRI expresses AST relative to its upper limit of normal [11,12]. These differences may affect their associations with clinical outcomes and make their comparison relevant in an NSTEMI population. Age may contribute to the association of FIB-4 with renal outcomes, while transaminase levels during an acute coronary event may be influenced by myocardial injury, congestion, or hypoperfusion. An elevated score may therefore reflect both chronic patient characteristics and acute illness, rather than hepatic fibrosis alone. This possibility is relevant to covariate adjustment and to the interpretation of any association observed in an acute-care cohort.

Evidence linking these indices to renal outcomes also warrants consideration, although it comes from different clinical settings. In a longitudinal study of Japanese men, higher FIB-4 was associated with incident chronic kidney disease and declining estimated glomerular filtration rate in selected metabolically healthy subgroups; the association with incident chronic kidney disease was not statistically significant in the overall propensity-matched population [15]. Among patients undergoing elective percutaneous coronary intervention, CA-AKI has been linked to several liver fibrosis scores, FIB-4 and APRI among them [16]. In acute coronary syndromes, contrast-induced nephropathy was more frequent among patients with high FIB-4 values, although this observation was unadjusted and derived from a mixed cohort [17]. These findings provide a basis for further investigation, but neither chronic renal outcomes in a health-screening population, results from elective coronary procedures, nor unadjusted data from mixed acute coronary syndrome cohorts can be assumed to apply directly to NSTEMI. The acute clinical context may alter both the components of the indices and their relationship with subsequent kidney injury.

Accordingly, this study examined the associations of admission FIB-4 and APRI with CA-AKI in patients with NSTEMI undergoing coronary angiography. We compared their discrimination and explored whether adding FIB-4 to the Mehran score improved model performance. The objective was to assess the relevance of these routinely available composite indices in this setting, without assuming that they measure renal fibrosis or that an adjusted association necessarily translates into clinically useful prediction.

## 2. Materials and Methods

### 2.1. Study Design and Population

We retrospectively reviewed consecutive patients undergoing coronary angiography for NSTEMI at a tertiary cardiology center from May 2020 to May 2025. The initial screening population comprised 1053 patients.

Eligibility assessment excluded patients with documented chronic liver disease, active malignancy, current or previous hematological disease, dialysis-dependent end-stage renal disease, or active infection. Additional exclusions were death during the first 48 h of hospitalization, coronary artery bypass grafting (CABG) during the first 72 h, and insufficient data to calculate either fibrosis index or determine CA-AKI status. Patients who died within 48 h were excluded because the post-angiography creatinine assessment required for CA-AKI ascertainment could not be completed. Patients who underwent CABG within 72 h were excluded because perioperative changes in renal function would complicate interpretation of the post-angiography creatinine outcome. Overall, 282 of the 1053 screened patients were excluded, including 12 who died within 48 h and 33 who underwent CABG within 72 h, resulting in a study cohort of 771 patients.

### 2.2. Data Collection and Definitions

Information on demographics and conventional cardiovascular risk factors was extracted from electronic medical records and discharge summaries. These variables comprised age, sex, diabetes mellitus, hypertension, dyslipidemia, prior myocardial infarction, previous coronary revascularization, heart failure, chronic kidney disease, chronic obstructive pulmonary disease, smoking status, and medications at admission. Heart failure referred to a pre-existing diagnosis recorded in the medical history, rather than newly developed heart failure during the index NSTEMI admission. Historical left ventricular ejection fraction (LVEF) measurements were unavailable. Killip class was recorded at presentation.

Baseline laboratory data, measured in blood samples drawn on admission, comprised liver enzymes [AST and ALT], blood count parameters (platelet count, hemoglobin, and white blood cell count), renal markers [serum creatinine, estimated glomerular filtration rate (eGFR), and blood urea nitrogen], C-reactive protein CRP, fasting plasma glucose, and serum magnesium, potassium, and sodium. Serum creatinine at hospital admission was used as the baseline value. The highest available serum creatinine value measured between 48 and 72 h after coronary angiography was selected for outcome assessment. Serial high-sensitivity cardiac troponin T (hs-cTnT) measurements obtained during hospitalization were used to determine peak values. Glycated hemoglobin (HbA1c) and lipid parameters (total cholesterol, low-density lipoprotein cholesterol, and triglycerides) were obtained from the laboratory database of the hospital.

Every diagnostic and interventional coronary procedure used the same nonionic, low-osmolar contrast medium (iohexol, Omnipaque 350 mg/mL; GE Healthcare, Cork, Ireland). Total contrast volume for each procedure was retrieved from the digital angiography registry. There was no standardized hydration protocol; hydration decisions were individualized according to renal function and clinical status.

As part of routine care, transthoracic echocardiography was undertaken within 48 h of admission, and left ventricular ejection fraction was measured with the modified biplane Simpson method. Angiographic and procedural information was obtained from the institutional imaging archive. CA-AKI was operationally defined as an increase in serum creatinine of ≥0.3 mg/dL or an increase to ≥1.5 times the admission value, using the highest measurement obtained 48–72 h after coronary angiography. This definition used the Kidney Disease: Improving Global Outcomes (KDIGO) serum creatinine thresholds with an adapted assessment window, rather than strictly reproducing the KDIGO timing requirements [4]. Creatinine measurements beyond 72 h were not consistently available because many patients had been discharged. Urine output was not recorded and therefore could not be used as a criterion.

NSTEMI was defined according to the Fourth Universal Definition of Myocardial Infarction [18], consistent with the 2023 European Society of Cardiology guidelines on acute coronary syndromes [8].

The Mehran risk score was retrospectively calculated for research purposes from recorded clinical, laboratory, and procedural data according to the original model [7]. This research calculation was not used to guide patient management. Because the score includes total contrast volume, it was not treated as a fully available admission or pre-procedural risk assessment [7].

### 2.3. Calculation of Fibrosis-Based Indices

FIB-4 and APRI were computed with the formulas described in their original validation studies [11,12]:

• **FIB-4 index** = (Age [years] × AST [IU/L])/(Platelet count [×10^9^/L] × √ALT [IU/L])
• **APRI** = (AST [IU/L]/upper limit of normal AST [37 IU/L]) × 100/Platelet count [×10^9^/L]

### 2.4. Statistical Analysis

SPSS Statistics, version 27.0 (IBM Corp., Armonk, NY, USA), was used for the original analyses. The additional nested-model and receiver operating charactericstic (ROC)-curve comparisons were performed using Python version 3.12.14 (Python Software Foundation, Wilmington, DE, USA) with SciPy version 1.17.0 and scikit-learn version 1.8.0. Normality of continuous variables was examined with the Kolmogorov–Smirnov test and by inspecting skewness. Approximately normally distributed data are summarized as mean ± standard deviation (SD), and skewed data as median and interquartile range (IQR; 25th–75th percentiles). Categorical data are expressed as numbers and percentages.

For group comparisons, Welch’s *t*-test was applied to approximately normal continuous variables and the Mann–Whitney U test to skewed variables. The chi-square (χ^2^) test or, when expected cell counts were small, Fisher’s exact test was used for categorical variables. The Fisher–Freeman–Halton exact test was used to compare Killip class distributions because of sparse cell counts.

Covariates were selected on the basis of clinical relevance and established associations with CA-AKI, rather than solely on statistical significance in univariable comparisons. Diabetes mellitus, hemoglobin, baseline serum creatinine, left ventricular ejection fraction, and contrast volume were chosen to represent distinct clinical and procedural aspects of risk. Baseline serum creatinine was used in the primary models because it was the reference measurement for CA-AKI ascertainment. Given the number of CA-AKI events in the complete-case analysis, the number of model parameters was limited to reduce the risk of overfitting. FIB-4 was evaluated as a composite index using its established formula; age was therefore not entered separately in the primary FIB-4 model. Separate multivariable logistic regression models were constructed for FIB-4 and APRI, with all covariates entered simultaneously using the Enter method. Analyses used a complete-case approach, excluding patients with missing values for any variable included in the respective model. Multiple imputation was not performed. Associations are expressed as odds ratios (ORs) and 95% confidence intervals (CIs).

Two multivariable models were evaluated. Model 1 contained the FIB-4 index together with diabetes mellitus, hemoglobin, baseline serum creatinine, left ventricular ejection fraction, and contrast volume. Diabetes mellitus was entered as a binary variable (present or absent), and the remaining covariates as continuous variables. In Model 2, APRI was substituted for FIB-4, with the covariates unchanged. As a sensitivity analysis, the FIB-4 model was repeated with eGFR replacing baseline serum creatinine in the same complete-case population. For this analysis, eGFR was calculated for all patients from admission serum creatinine, age, and sex using the 2021 Chronic Kidney Disease Epidemiology Collaboration (CKD-EPI) creatinine equation [19]. An additional sensitivity analysis incorporated CRP into the primary FIB-4 model. Models with and without CRP were compared in the same complete-case population to distinguish the effect of CRP adjustment from differences in sample composition. Additional sensitivity analyses incorporated age, ALT, and both variables into the primary FIB-4 model while retaining the original adjustment covariates. Age and ALT were entered as continuous variables, and all models were fitted in the same complete-case population (Supplementary Tables S1 and S2). The extent and pattern of missing data and comparisons of available characteristics between patients included in and excluded from the primary models are presented in Supplementary Table S3.

Multicollinearity in both primary regression models was assessed using variance inflation factors (VIFs) and tolerance statistics. The primary models included six predictor parameters and 83 CA-AKI events, corresponding to 13.8 events per predictor parameter; the CRP-adjusted sensitivity model included seven predictor parameters and 82 events, corresponding to 11.7 events per predictor parameter, excluding the intercept. Nagelkerke R^2^ and −2 log-likelihood values were used to describe model fit. Receiver operating characteristic (ROC) curves were constructed to evaluate how well FIB-4 and APRI discriminated between patients with and without CA-AKI, and the area under the curve (AUC) is reported together with its 95% CI. Exploratory cut-off values were derived using the Youden index and evaluated in the same dataset; they were not independently validated. The AUCs of FIB-4 and APRI were compared using the paired DeLong test. At each identified cut-off, we calculated sensitivity, specificity, positive predictive value (PPV), and negative predictive value (NPV).

To assess the incremental predictive value of FIB-4 beyond an established risk score, we compared two nested logistic regression models: one containing the Mehran risk score alone and the other containing the Mehran risk score and FIB-4. Both models were fitted in the same patients with complete data for these variables. Their areas under the receiver operating characteristic curve (AUCs) were compared using a paired DeLong test. A likelihood-ratio test was used to assess the change in model fit after adding FIB-4. This additional analysis was exploratory, and its performance estimates were derived from the study cohort without external validation. Because age is a component of FIB-4 and only partly represented in the Mehran score, the incremental analysis was repeated after adding age to the Mehran-score model. Calibration of the primary FIB-4 model and of the Mehran-based models was assessed with the Hosmer–Lemeshow test across deciles of predicted risk and with the Brier score, and the calibration slope was estimated using 500 bootstrap resamples. As a renal function-normalized measure of contrast exposure, the ratio of contrast volume to eGFR was also examined.

Two-sided *p*-values below 0.05 indicated statistical significance.

## 3. Results

The final analysis comprised 771 patients with NSTEMI undergoing coronary angiography, of whom 97 (12.6%) developed CA-AKI.

Table 1 summarizes baseline demographic, clinical, laboratory, and procedural characteristics by CA-AKI status. Compared with patients without CA-AKI, those who developed it were significantly older and more often had chronic obstructive pulmonary disease, prior stroke, and heart failure (*p* < 0.05 for all); the distribution of diabetes treatment categories also differed between groups (*p* = 0.021). Chronic kidney disease was also more common in the CA-AKI (+) group, although this difference was of borderline significance (*p* = 0.056). No significant between-group differences were found for sex, body mass index, hypertension, hyperlipidemia, prior myocardial infarction, previous coronary angiography or revascularization, or medical therapy at admission. Among the 17 patients with a history of heart failure, LVEF measured within the first 48 h of admission was <50% in 14 patients and ≥50% in 1 patient; LVEF was unavailable in 2 patients.

Among laboratory and echocardiographic variables, the CA-AKI group had significantly lower left ventricular ejection fraction and hemoglobin and higher blood urea nitrogen (all *p* < 0.05). This group also had higher baseline serum creatinine (*p* = 0.004) and lower baseline eGFR (*p* < 0.001). CRP was higher (*p* = 0.034) and ALT lower (*p* = 0.017) in patients who developed CA-AKI. Peak hs-cTnT tended to be higher in the CA-AKI group, but the difference was not statistically significant (*p* = 0.071). As expected, creatinine at 48–72 h was considerably higher in patients with CA-AKI (*p* < 0.001). The Mehran risk score and FIB-4 index were both significantly higher in the CA-AKI group (both *p* < 0.001), whereas APRI values were similar between groups (*p* = 0.261) (Table 1).

Patients with CA-AKI also had a higher Killip class distribution and a longer hospital stay (*p* = 0.006 and *p* = 0.001, respectively). Contrast volume and revascularization strategy did not differ between groups, whereas in-hospital dialysis was required significantly more often in the CA-AKI (+) group (*p* = 0.016). Most patients (58.5%) received exactly 100 mL of contrast, with similar proportions in patients with and without CA-AKI (54.7% and 59.0%, respectively); however, the contrast volume-to-eGFR ratio was higher in patients who developed CA-AKI (median 1.81 versus 1.54; *p* = 0.018).

Missing covariate data comprised LVEF in 92 patients (11.93%), diabetes mellitus status in 3 (0.39%), and contrast volume in 3 (0.39%). One patient lacked both LVEF and diabetes mellitus data, resulting in 97 unique patients excluded from the complete-case analyses. FIB-4, APRI, hemoglobin, and baseline creatinine were available for all 771 patients. No statistically significant differences were observed in the available characteristics compared between patients included in and excluded from the primary models, including CA-AKI frequency (12.3% versus 14.4%; *p* = 0.516). The extent and pattern of missing data for variables used in the primary and sensitivity models and the included-versus-excluded comparisons are presented in Supplementary Table S3.

Table 2 shows the multivariable logistic regression results for the 674 patients with complete covariate data, 83 of whom developed CA-AKI. FIB-4 was independently associated with CA-AKI in Model 1 (OR 1.19, 95% CI 1.04–1.36; *p* = 0.013), as were LVEF (OR 0.96, 95% CI 0.94–0.98; *p* < 0.001) and hemoglobin (OR 0.86, 95% CI 0.75–0.98; *p* = 0.021). In Model 2, APRI showed no significant association with CA-AKI (OR 1.02, 95% CI 0.65–1.60; *p* = 0.921), whereas LVEF (OR 0.95, 95% CI 0.93–0.97; *p* < 0.001) and hemoglobin (OR 0.85, 95% CI 0.75–0.97; *p* = 0.018) remained independently associated. Model 1 had a Nagelkerke R^2^ of 0.102 and a −2 log-likelihood of 465.715; the corresponding values for Model 2 were 0.087 and 471.589. VIFs ranged from 1.02 to 1.10 in both primary models, with tolerance values above 0.90, indicating no problematic multicollinearity.

When baseline serum creatinine was replaced with eGFR in the same 674 patients, including 83 CA-AKI events, FIB-4 remained associated with CA-AKI (OR 1.18, 95% CI 1.02–1.35; *p* = 0.022; Supplementary Table S1). In a sensitivity analysis including 653 patients with complete data and 82 CA-AKI events, FIB-4 remained associated with CA-AKI after additional adjustment for CRP (OR 1.18, 95% CI 1.03–1.36; *p* = 0.019; Supplementary Table S1). In additional sensitivity analyses involving the same 674 patients and 83 CA-AKI events, the association of FIB-4 with CA-AKI was attenuated after adjustment for age alone (OR 1.13, 95% CI 0.98–1.31; *p* = 0.092), but remained statistically significant after adjustment for ALT alone (OR 1.27, 95% CI 1.09–1.47; *p* = 0.002) and for both age and ALT (OR 1.20, 95% CI 1.03–1.40; *p* = 0.018). In the model incorporating both variables, variance inflation factors ranged from 1.02 to 1.27, with 10.4 events per predictor parameter, excluding the intercept (Supplementary Table S2). When contrast volume and baseline creatinine were replaced by the contrast volume-to-eGFR ratio, FIB-4 remained associated with CA-AKI (OR 1.18, 95% CI 1.03–1.35; *p* = 0.017), whereas the ratio was not independently associated with the outcome (OR 1.10, 95% CI 0.96–1.27; *p* = 0.176).

**Figure 1:**
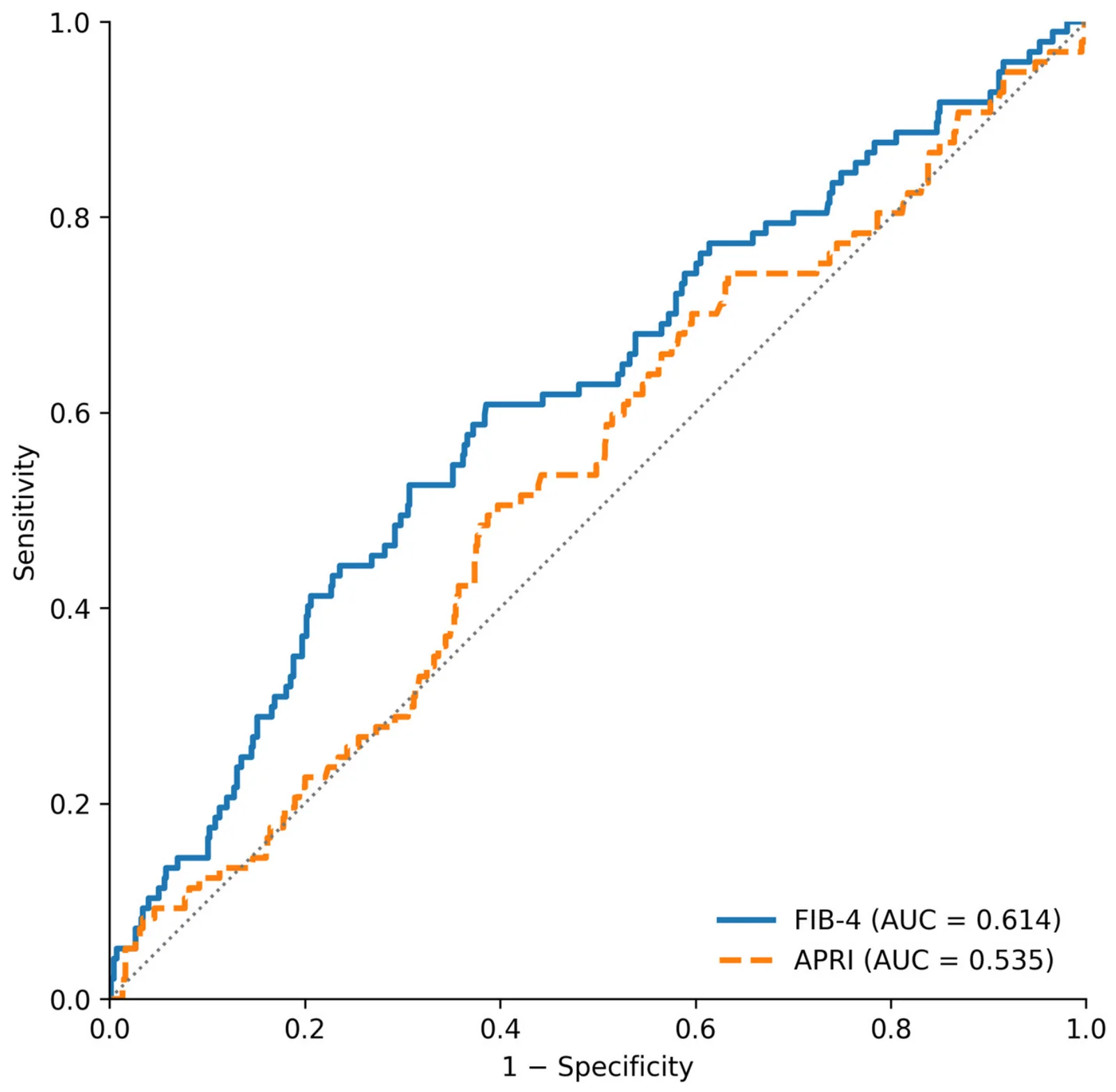
ROC curves for FIB-4 and APRI in relation to CA-AKI. FIB-4 had a significantly higher AUC than APRI (paired DeLong *p* < 0.001). The solid and dashed lines represent FIB-4 and APRI, respectively, and the dotted diagonal line indicate no discrimination (AUC = 0.5).

ROC analysis showed higher discrimination for FIB-4 than for APRI (Table 3, Figure 1).

FIB-4 had an AUC of 0.614 (95% CI 0.552–0.676), compared with 0.535 (95% CI 0.474–0.597) for APRI. The AUC of FIB-4 was significantly higher than that of APRI (AUC difference 0.078, 95% CI 0.044–0.113; paired DeLong *p* < 0.001). Using the exploratory Youden-derived cut-off of 1.44, FIB-4 had a sensitivity of 60.82%, a specificity of 61.42%, a PPV of 18.50%, and an NPV of 91.59%. For APRI, the exploratory, Youden-derived cut-off was 0.23, with a sensitivity of 74.23% and specificity of 36.65%.

In an exploratory incremental-value analysis, Mehran risk score data were available for 766 patients, including 93 who developed CA-AKI. In this same subset, the AUC was 0.646 for the model containing the Mehran score alone and 0.666 for the model containing the Mehran score and FIB-4 (absolute difference, 0.020). The paired DeLong test did not show a statistically significant difference between the AUCs (95% CI for the difference, −0.004 to 0.044; *p* = 0.100). Adding FIB-4 improved model fit according to the likelihood-ratio test (*p* = 0.009). Thus, the improvement in model fit was not accompanied by a clearly established improvement in discrimination (Table 4). When age was added to the Mehran-score model, FIB-4 remained associated with CA-AKI (OR 1.17, 95% CI 1.02–1.33; *p* = 0.021) and improved model fit (likelihood-ratio *p* = 0.025), whereas the increase in AUC from 0.674 to 0.684 was not statistically significant (difference 0.010, 95% CI −0.008 to 0.029; DeLong *p* = 0.282). The primary FIB-4 model showed no evidence of miscalibration (Hosmer–Lemeshow *p* = 0.141; Brier score 0.100), and its bootstrap calibration slope of 0.90 indicated modest optimism (Supplementary Table S4).

## 4. Discussion

In this cohort of patients with NSTEMI undergoing coronary angiography, FIB-4 was associated with CA-AKI after adjustment for the covariates included in the primary model, whereas APRI was not. FIB-4 also showed significantly higher discrimination than APRI in the paired comparison. These findings extend previous observations concerning the cardiovascular relevance of FIB-4 to an early renal outcome in an acute coronary population. By examining both the adjusted association and performance beyond the Mehran score, the study provides a more complete assessment of its potential role in renal risk evaluation. The results support further investigation of FIB-4 as a readily available adjunct, while its modest discrimination requires a distinction between an associated marker and a clinically useful prediction tool. To our knowledge, this is the first study to evaluate the adjusted association between FIB-4 and CA-AKI specifically in patients with NSTEMI, comparing it directly with APRI and examining its value beyond the Mehran score. The association persisted in four of the five sensitivity analyses, including adjustment for CRP, replacement of baseline creatinine with eGFR, and simultaneous adjustment for age and ALT.

The clinical profile of patients who developed CA-AKI suggests that renal deterioration occurred in the setting of several coexisting risk factors. These patients were older and had lower LVEF and poorer baseline renal function; heart failure and cerebrovascular disease were also more frequent. Contrast volume and revascularization strategies did not differ significantly between outcome groups. This pattern is consistent with the importance of baseline renal function and cardiovascular status described in coronary intervention cohorts [5,7,9]. Because AKI after angiography may also be influenced by haemodynamic compromise, myocardial injury, inflammation, medications, and baseline cardiorenal vulnerability, its occurrence after contrast exposure does not establish contrast as the sole cause. Prevention therefore requires attention to the patient’s overall clinical and renal risk. Although baseline creatinine was higher in patients with CA-AKI, it was not independently associated with the outcome; its estimate moved toward the null mainly after adjustment for LVEF and hemoglobin, and the wide confidence interval (OR 0.91, 95% CI 0.45–1.84) reflects shared information with these covariates within a narrow creatinine range rather than a protective effect.

Laboratory-based composite indices have been investigated as a means of summarizing additional aspects of risk in acute coronary syndromes. Çiçek et al. reported an independent association between the pan-immune-inflammation value and CA-AKI in patients with ST-segment elevation myocardial infarction (STEMI) undergoing primary percutaneous coronary intervention [20]. This supports interest in routinely available laboratory indices, although differences in clinical presentation and procedural urgency limit direct extrapolation to NSTEMI. Their evaluation should complement attention to established clinical risk factors and preventive care. A systematic review and meta-analysis by Dimitriadis et al. reported an association between chronic sodium–glucose cotransporter 2 (SGLT2) inhibitor treatment and lower post-procedural AKI risk in patients with type 2 diabetes undergoing coronary procedures; however, these findings should not be extrapolated to all patients with NSTEMI or interpreted as evidence that SGLT2 inhibitor initiation prevents CA-AKI in this cohort [21].

The prognostic value of FIB-4 in acute coronary syndromes has been reported previously, with higher values linked to adverse cardiovascular events and death [13,14]. However, the interpretation of FIB-4 in NSTEMI requires consideration of the circumstances in which its components were measured. As noted in the Introduction, the two indices differ in that FIB-4 additionally includes age and ALT [11,12]. Their differing associations with CA-AKI may therefore reflect differences in the information combined by the formulas, rather than differences in hepatic fibrosis.

Admission transaminase levels are particularly relevant to this interpretation. In a prospective study of patients with acute coronary syndromes, Jasiewicz et al. documented frequent AST and ALT elevations during the first 24 h, with marked AST elevations common among patients with more extensive myocardial injury [22]. This illustrates why transaminase abnormalities in an acute coronary setting cannot automatically be attributed to chronic liver disease. In our cohort, ALT was lower in patients who developed CA-AKI. Because ALT appears in the denominator of FIB-4, lower values can increase the index even without a corresponding increase in AST or a reduction in platelet count. This mathematical contribution does not identify the clinical reason for the lower ALT values. Together with the possible effects of myocardial injury, congestion, and hypoperfusion on transaminase measurements, it supports interpreting admission FIB-4 as a composite of chronic patient characteristics and acute physiological disturbances rather than as a direct estimate of fibrosis.

The sensitivity analyses provided additional information about the stability and interpretation of the primary finding. The association persisted after adjustment for CRP and after simultaneous adjustment for age and ALT, suggesting that it was not fully accounted for by these measured variables in the corresponding models. However, adjustment for age alone attenuated the association to statistical non-significance. Age-related variation in FIB-4 performance has also been reported in liver fibrosis assessment, underscoring the importance of interpreting the index in relation to patient age [23]. The association also persisted when baseline creatinine was replaced with eGFR calculated for all patients, although the CKD-EPI equation itself incorporates age. Taken together, these findings support the association under several adjustment strategies but show that it was not consistent across all model specifications, with adjustment for age alone producing the principal attenuation. They also indicate that the information conveyed by FIB-4 cannot be cleanly separated from its constituent variables or interpreted as evidence of a specific fibrosis-mediated mechanism.

Although the prognostic significance of FIB-4 has been studied more extensively, data directly linking FIB-4 to CA-AKI remain limited and derive mainly from elective procedures or from unadjusted comparisons in mixed acute coronary syndrome cohorts. Associations between liver fibrosis-based indices and CA-AKI have also been described after elective endovascular repair of abdominal aortic aneurysms, a relatively stable procedural population [24]. Likewise, in a large retrospective cohort with stable coronary artery disease treated with elective percutaneous coronary intervention, several non-invasive liver fibrosis scores, FIB-4 and APRI among them, were independently related to CA-AKI, although each score discriminated only modestly [16]. Unlike the elective PCI study, APRI was not associated with CA-AKI in our adjusted analysis. Differences in patient selection, outcome definitions, and covariate adjustment may contribute to this discrepancy. In an acute coronary syndrome cohort of 1465 patients undergoing coronary angiography or PCI, Sielski et al. observed the highest incidence of contrast-induced nephropathy among patients with FIB-4 ≥ 3.25 (10.0% versus 4.8% with FIB-4 < 1.45); however, this comparison was unadjusted, used a different nephropathy definition, and the multivariable analyses of that study addressed mortality rather than renal outcomes [17]. The present study adds adjusted evidence restricted to an NSTEMI population, together with a direct comparison with APRI and an assessment beyond the Mehran score, in a setting where acute myocardial injury and haemodynamic disturbances may affect both the index components and renal outcomes. This distinction makes the acute coronary setting a relevant and so far under-investigated context for further evaluation of these scores.

Notably, absolute contrast volume was not independently associated with CA-AKI in this study, although its distribution was narrow, with 58.5% of patients receiving exactly 100 mL. Although contrast dose is incorporated into traditional risk assessment and remains a potentially modifiable procedural factor [4,7,25], the absence of an association in this cohort should not be interpreted as evidence that contrast exposure is free of renal risk. This may be explained by the range of contrast exposure, by patient selection, or by confounding. A further consideration is that absolute contrast volume does not express exposure relative to renal clearance. Laskey et al. demonstrated an association between the contrast volume-to-creatinine clearance ratio and an early rise in serum creatinine after PCI [26]. This suggests that similar administered volumes may have different implications in patients with different baseline renal function. In our cohort, the contrast volume-to-eGFR ratio was higher in patients who developed CA-AKI but was not independently associated with the outcome after multivariable adjustment, and FIB-4 remained associated in this model. The narrow range of administered volumes limits the ability of this study to assess dose-dependent contrast effects. Future studies could therefore examine whether renal function-normalized contrast measures modify the relationship between FIB-4 and CA-AKI.

FIB-4 has also been linked to renal outcomes outside the setting of contrast exposure. Beyond the subgroup-limited association with incident chronic kidney disease in a health-screening cohort noted in the Introduction [15], FIB-4 has also been examined in acute heart failure. Irie et al. reported that higher admission FIB-4 was associated with improved renal function during hospitalization in patients with acute heart failure and LVEF ≥ 40%, with improvement defined as a ≥20% increase in eGFR [27]. This renal phenotype was nevertheless associated with poorer subsequent survival and was discussed in relation to congestion. These contrasting observations suggest that the relationship between FIB-4 and renal function depends on the clinical setting and outcome assessed. Together with the present findings, they broaden the rationale for renal research involving FIB-4 across different clinical settings, in which the underlying mechanisms may differ.

The practical appeal of FIB-4 lies in its calculation from age and routinely measured laboratory variables, allowing evaluation without an additional biomarker assay. In this study, adding FIB-4 to the Mehran score improved model fit, providing a reason to investigate whether it captures information that may contribute to a combined assessment. The absolute increase in AUC was nevertheless small, and the paired comparison did not establish a statistically significant improvement in discrimination. The improvement in model fit nevertheless indicates that FIB-4 carries information not fully captured by the Mehran score, which warrants evaluation in larger cohorts. Because the original Mehran score includes procedural contrast volume, the present analysis also does not resolve whether admission FIB-4 adds useful information to a model based entirely on pre-angiography variables.

The threshold was derived and evaluated in the same cohort and should therefore be regarded as exploratory; its interpretation also requires consideration of the modest standalone discrimination and the dependence of predictive values on outcome prevalence. At a CA-AKI prevalence of 12.6%, the high NPV does not establish a reliable rule-out strategy, while the PPV of 18.5% and sensitivity of 60.8% limit identification of individual events. The more promising direction for further research may be to evaluate FIB-4 as a continuous component of a broader clinical model rather than as a dichotomous decision threshold. Independent validation should establish whether its contribution is reproducible, improves calibration, and provides benefit for a defined clinical purpose. The present findings justify this investigation but do not yet support using FIB-4 alone to direct clinical care.

This study has several limitations. Its retrospective, single-center design introduces a risk of selection bias and limits generalizability. Excluding patients who died within 48 h or underwent CABG within 72 h may have removed patients with a more severe early clinical course, further limiting applicability to these groups. CA-AKI assessment used KDIGO serum creatinine thresholds within an adapted 48–72 h post-angiography window; this did not ensure that the absolute increase occurred within 48 h, and later events could not be assessed consistently, potentially contributing to outcome misclassification. Data on angiography timing and urgency, hydration volume and duration, nephrotoxic medication use or management, and specific preventive measures were unavailable, leaving potential residual confounding. Patients undergoing urgent procedures may have had less opportunity for pre-procedural hydration and greater clinical instability. Historical LVEF measurements and separate assessments of congestion or haemodynamic instability immediately before angiography were also unavailable, limiting characterization of pre-existing heart failure. FIB-4 and APRI may have been affected by transient biochemical changes during the acute coronary event, and the sensitivity analyses did not establish independence from all FIB-4 components. Complete-case analyses may have introduced missing-data bias. The incremental-performance analysis and exploratory threshold were evaluated in the same cohort used for model fitting or threshold selection, so their apparent performance may be optimistic and requires independent validation. Finally, the lack of long-term follow-up precluded assessment of renal recovery and outcomes beyond the index hospitalization.

## 5. Conclusions

In patients with NSTEMI undergoing coronary angiography, FIB-4 was associated with CA-AKI in the primary adjusted model, whereas APRI was not. FIB-4 showed higher discrimination than APRI, although its standalone performance was modest and its association was sensitive to covariate adjustment, particularly for age. Adding FIB-4 to the Mehran score improved model fit without establishing a significant improvement in discrimination. Further studies are warranted to determine whether FIB-4 can serve as a useful adjunct to renal risk assessment, and independent validation is required to establish its incremental clinical value.

## Figures and Tables

**Table 1:** Baseline demographic, laboratory and procedural characteristics according to CA-AKI status.

Variable	CA-AKI (–) (*n* = 674)	CA-AKI (+) (*n* = 97)	*p*-Value
Demographic characteristics			
Age, years	59.15 ± 11.22	65.90 ± 11.56	<0.001 *
Sex, female	193 (28.6%)	29 (29.9%)	0.797
Body mass index, kg/m^2^	28.52 (25.91–31.71)	27.95 (25.76–31.93)	0.704
Hypertension	361 (53.6%)	57 (58.8%)	0.346
Diabetes mellitus	230 (34.3%)	43 (44.3%)	0.021 *
Insulin-treated DM	99 (14.8%)	25 (25.8%)	
Oral antidiabetic use	131 (19.5%)	18 (18.6%)	
Chronic obstructive pulmonary disease	29 (4.3%)	9 (9.3%)	0.046 *
Peripheral arterial disease	37 (5.5%)	6 (6.2%)	0.797
Stroke history	22 (3.3%)	8 (8.2%)	0.042 *
History of heart failure	11 (1.7%)	6 (6.5%)	0.014 *
Chronic kidney disease	36 (5.3%)	10 (10.3%)	0.056
Hyperlipidemia	81 (12.0%)	10 (10.3%)	0.626
Previous myocardial infarction	169 (25.1%)	24 (24.7%)	0.838
Prior coronary angiography	213 (31.6%)	35 (36.1%)	0.437
Previous revascularization			0.914
CABG	28 (4.2%)	4 (4.1%)	
PCI	136 (20.2%)	17 (17.5%)	
PCI + CABG	16 (2.4%)	3 (3.1%)	
Medical treatment at admission			
ACEi/ARB	245 (36.4%)	35 (36.1%)	0.963
Statin	121 (18.0%)	18 (18.6%)	0.822
Laboratory parameters			
LVEF, %	52.26 ± 9.55	46.65 ± 11.39	<0.001 *
CRP, mg/dL	0.60 (0.20–1.60)	1.00 (0.30–2.08)	0.034 *
WBC, ×10^3^/µL	9.40 (7.88–11.40)	9.80 (7.60–12.00)	0.639
Hemoglobin, g/dL	14.04 ± 1.83	13.53 ± 1.96	0.018 *
Platelet count, ×10^3^/µL	230 (196–268)	232 (185–273)	0.667
Blood urea nitrogen, mg/dL	16 (13–20)	18 (14–24)	0.006 *
Baseline eGFR, mL/min/1.73 m^2^	88.57 ± 20.91	78.76 ± 23.33	<0.001 *
Baseline creatinine, mg/dL	0.85 (0.77–1.02)	0.93 (0.81–1.13)	0.004 *
Creatinine at 48–72 h, mg/dL	0.87 (0.79–1.02)	1.34 (1.12–1.70)	<0.001 *
Peak hs-cTnT, ng/mL	0.70 (0.08–4.26)	1.04 (0.14–11.55)	0.071
Glucose, mg/dL	116 (100–170)	117 (100–183)	0.926
HbA1c, %	6.10 (5.70–7.30)	6.20 (5.70–7.50)	0.353
Total cholesterol, mg/dL	190.89 ± 45.64	198.55 ± 51.08	0.173
LDL cholesterol, mg/dL	118.34 ± 38.45	128.49 ± 45.24	0.046 *
Triglycerides, mg/dL	155 (109–230)	156 (117–238)	0.468
Sodium, mmol/L	139.47 ± 2.66	138.99 ± 2.89	0.126
Potassium, mmol/L	4.36 ± 0.44	4.42 ± 0.64	0.375
Magnesium, mg/dL	1.98 (1.80–2.10)	1.98 (1.83–2.20)	0.709
AST, U/L	23 (18–33)	24 (18–33)	0.326
ALT, U/L	22 (16–32)	20 (15–27)	0.017 *
Mehran risk score	3 (1–6)	5 (2–9)	<0.001 *
APRI	0.27 (0.20–0.42)	0.30 (0.21–0.42)	0.261
FIB-4 index	1.28 (0.92–1.85)	1.63 (1.11–2.48)	<0.001 *
Clinical and procedural characteristics			
Killip class, *n* (%)			0.006 *
I	581 (97.0%)	78 (89.7%)	
II	5 (0.8%)	2 (2.3%)	
III	13 (2.2%)	7 (8.0%)	
Revascularization			0.529
None	229 (33.9%)	37 (38.1%)	
PCI	319 (47.3%)	46 (47.4%)	
CABG	126 (18.7%)	14 (14.4%)	
Multivessel PCI at index procedure	39 (5.8%)	4 (4.1%)	0.569
Staged PCI	30 (4.5%)	6 (6.2%)	0.426
Contrast volume, mL	100 (100–200)	100 (100–200)	0.751
Length of hospital stay, days	6 (4–10)	8 (5–13)	0.001 *
Dialysis in hospital	2 (0.3%)	3 (3.1%)	0.016 *
In-hospital mortality	3 (0.4%)	0 (0.0%)	1.000

Values are mean ± standard deviation, median (interquartile range [25th–75th percentiles]), or n (%). Groups were compared with Welch’s *t*-test for approximately normal continuous variables, the Mann–Whitney U test for skewed continuous variables, and the chi-square or Fisher’s exact test for categorical variables. Killip class distributions were compared using the Fisher–Freeman–Halton exact test. Percentages for diabetes mellitus, history of heart failure, and Killip class were calculated using available observations. Baseline eGFR was calculated for all patients using the 2021 CKD-EPI creatinine equation. * *p* < 0.05. The diabetes *p*-value compares no diabetes, insulin use with or without oral antidiabetics, and oral antidiabetic use alone. ACEi: angiotensin-converting enzyme inhibitor; ALT: alanine aminotransferase; APRI: AST-to-platelet ratio index; ARB: angiotensin receptor blocker; AST: aspartate aminotransferase; CA-AKI: contrast-associated acute kidney injury; CABG: coronary artery bypass grafting; CKD-EPI: Chronic Kidney Disease Epidemiology Collaboration; CRP: C-reactive protein; DM: diabetes mellitus; eGFR: estimated glomerular filtration rate; FIB-4: fibrosis-4 index; HbA1c: glycated hemoglobin; hs-cTnT: high-sensitivity cardiac troponin T; LDL: low-density lipoprotein; LVEF: left ventricular ejection fraction; PCI: percutaneous coronary intervention; WBC: white blood cell count.

**Table 2:** Multivariable logistic regression analysis for factors associated with CA-AKI.

Variable	Model 1 OR (95% CI)	*p*-Value	Model 2 OR (95% CI)	*p*-Value
Hemoglobin (g/dL)	0.86 (0.75–0.98)	0.021 *	0.85 (0.75–0.97)	0.018 *
Diabetes mellitus	1.15 (0.69–1.90)	0.595	1.12 (0.68–1.86)	0.647
LVEF (%)	0.96 (0.94–0.98)	<0.001 *	0.95 (0.93–0.97)	<0.001 *
FIB-4 index	1.19 (1.04–1.36)	0.013 *	—	—
Baseline creatinine (mg/dL)	0.91 (0.45–1.84)	0.794	0.99 (0.50–1.94)	0.970
Contrast volume (mL)	1.002 (0.999–1.004)	0.199	1.002 (0.999–1.004)	0.206
APRI	—	—	1.02 (0.65–1.60)	0.921

Data are odds ratios (ORs) with 95% confidence intervals (CIs) from binary logistic regression with simultaneous entry of all covariates (Enter method); diabetes mellitus was entered as a binary variable. Nagelkerke R^2^: 0.102 (Model 1) and 0.087 (Model 2); −2 log-likelihood: 465.715 and 471.589, respectively. * *p* < 0.05 was considered statistically significant. CA-AKI: contrast-associated acute kidney injury; LVEF: left ventricular ejection fraction; APRI: AST-to-platelet ratio index; FIB-4: fibrosis-4 index.

**Table 3:** ROC analysis of FIB-4 and APRI for CA-AKI.

Score	Exploratory Cut-Off Value	Sensitivity (%)	Specificity (%)	PPV (%)	NPV (%)	AUC (95% CI)
APRI	0.23	74.23	36.65	14.43	90.81	0.535 (0.474–0.597)
FIB-4	1.44	60.82	61.42	18.50	91.59	0.614 (0.552–0.676)

ROC: receiver operating characteristic; AUC: area under the curve; PPV: positive predictive value; NPV: negative predictive value; CA-AKI: contrast-associated acute kidney injury; APRI: AST-to-platelet ratio index; FIB-4: fibrosis-4 index.

**Table 4:** Incremental discrimination after adding FIB-4 to the Mehran risk score.

Model	Patients, *n*	CA-AKI Events, *n*	AUC
Mehran risk score	766	93	0.646
Mehran risk score + FIB-4	766	93	0.666

Table note: The absolute AUC difference was 0.020 (95% CI, −0.004 to 0.044; paired DeLong *p* = 0.100). The likelihood-ratio test comparing the nested models yielded *p* = 0.009. AUC: area under the curve; CA-AKI: contrast-associated acute kidney injury; FIB-4: fibrosis-4 index.

## Data Availability

The data supporting the findings of this study are available from the corresponding author upon reasonable request. The data are not publicly available owing to patient privacy and institutional restrictions.

## Supplementary Materials

The following supporting information can be downloaded at https://www.mdpi.com/article/10.3390/jcm15218594/s1, Table S1. Sensitivity analyses of the association of FIB-4 with CA-AKI after additional adjustment for CRP and after replacement of baseline serum creatinine with eGFR; Table S2. Sensitivity analyses of the association between FIB-4 and CA-AKI after additional adjustment for age and ALT; Table S3. Extent and pattern of missing data and comparison of patients included in and excluded from the primary complete-case analyses; Table S4. Calibration and incremental performance of FIB-4-based models.
