# Supplementary material for: Association of FIB-4 and APRI with Contrast-Associated Acute Kidney Injury in Patients with Non-ST-Segment Elevation Myocardial Infarction Undergoing Coronary Angiography
Source: J Clin Med. 2026 Nov 4;15(21):8594. doi: 10.3390/jcm15218594 (PMC13764010; doi:10.3390/jcm15218594)
Supplement: Supplementary file 1 [file jcm-15-08594-s001.zip › jcm-4576081-supplementary.pdf]

Supplementary Table S1. Sensitivity analyses of the association of FIB-4 with CA-AKI after additional adjustment for CRP and after replacement of baseline serum creatinine with eGFR.

A. Additional adjustment for CRP

Model	Patients, n	CA-AKI events, n	FIB-4 OR (95% CI)	p-value
Primary covariates without CRP	653	82	1.19 (1.03–1.36)	0.015
Primary covariates with CRP	653	82	1.18 (1.03–1.36)	0.019

Both models included FIB-4, diabetes mellitus, hemoglobin, baseline serum creatinine, LVEF, and contrast volume. The second model additionally included CRP. FIB-4 and CRP were entered as continuous variables. Variance inflation factors in the CRP-adjusted model ranged from 1.03 to 1.10. Abbreviations: CA-AKI, contrast-associated acute kidney injury; CI, confidence interval; CRP, C-reactive protein; LVEF, left ventricular ejection fraction; OR, odds ratio.

B. Replacement of baseline serum creatinine with eGFR

Model	Patients, n	CA-AKI events, n	FIB-4 OR (95% CI)	p-value
Primary model (baseline serum creatinine)	674	83	1.19 (1.04–1.36)	0.013
Baseline serum creatinine replaced with eGFR	674	83	1.18 (1.02–1.35)	0.022

Both models included FIB-4, diabetes mellitus, hemoglobin, LVEF, and contrast volume, together with either baseline serum creatinine or eGFR, and were fitted in the same complete-case population. eGFR was calculated for all patients from the admission serum creatinine value used as the CA-AKI baseline, age, and sex using the 2021 CKD-EPI creatinine equation. Because this equation incorporates age, a component of FIB-4, this analysis partly overlaps with the age-adjusted analysis in Supplementary Table S2. Use of the 2009 CKD-EPI equation yielded a similar estimate (OR 1.17, 95% CI 1.02–1.35; p = 0.023). Variance inflation factors in the eGFR-adjusted model ranged from 1.03 to 1.27. FIB-4 and eGFR were entered as continuous variables. Abbreviations: CA-AKI, contrast-associated acute kidney injury; CI, confidence interval; CKD-EPI, Chronic Kidney Disease Epidemiology Collaboration; eGFR, estimated glomerular filtration rate; LVEF, left ventricular ejection fraction; OR, odds ratio.

Supplementary Table S2. Sensitivity analyses of the association between FIB-4 and CA-AKI after additional adjustment for age and ALT.

Model	Patients, n	CA-AKI events, n	FIB-4 OR (95% CI)	p-value
Primary model	674	83	1.19 (1.04–1.36)	0.013
Primary model + age	674	83	1.13 (0.98–1.31)	0.092
Primary model + ALT	674	83	1.27 (1.09–1.47)	0.002
Primary model + age + ALT	674	83	1.20 (1.03–1.40)	0.018

All models included FIB-4, diabetes mellitus, hemoglobin, baseline serum creatinine, LVEF, and contrast volume. Age and ALT were additionally included as indicated and were entered as continuous variables. Odds ratios for FIB-4 are expressed per 1-unit increase. All models used the same complete-case population. Abbreviations: ALT, alanine aminotransferase; CA-AKI, contrast-associated acute kidney injury; CI, confidence interval; LVEF, left ventricular ejection fraction; OR, odds ratio.

Supplementary Table S3. Extent and pattern of missing data and comparison of patients included in and excluded from the primary complete-case analyses.

A. Missing data for candidate variables considered in the primary and sensitivity models

Variable	Missing, n (%)
LVEF	92 (11.93)
CRP	27 (3.50)
Mehran score	5 (0.65)
Diabetes mellitus	3 (0.39)
Contrast volume	3 (0.39)
Age	0 (0.00)
Hemoglobin	0 (0.00)
Admission serum creatinine	0 (0.00)
eGFR (calculated)	0 (0.00)
AST	0 (0.00)
ALT	0 (0.00)
Platelet count	0 (0.00)
FIB-4	0 (0.00)
APRI	0 (0.00)
CA-AKI outcome	0 (0.00)

B. Available characteristics of patients included in and excluded from the primary models

Variable	Included (n = 674)	Excluded (n = 97)	p-value
Age, years	60 (52–69)	59 (53–65)	0.306
Diabetes mellitus, n/available n (%)	236/674 (35.0)	37/94 (39.4)	0.422
Hemoglobin, g/dL	14.2 (12.9–15.3)	14.1 (12.9–15.0)	0.532
Admission serum creatinine, mg/dL	0.86 (0.77–1.03)	0.87 (0.79–1.04)	0.793
eGFR, mL/min/1.73 m^2	94.0 (74.2–103.3)	93.3 (74.1–101.0)	0.746

Variable	Included (n = 674)	Excluded (n = 97)	p-value
Contrast volume, mL	100 (100–200)	100 (100–237.5)	0.200
FIB-4	1.31 (0.94–1.94)	1.19 (0.93–1.85)	0.414
APRI	0.28 (0.20–0.42)	0.26 (0.20–0.39)	0.454
CA-AKI, n/available n (%)	83/674 (12.3)	14/97 (14.4)	0.516

Values are presented as median (interquartile range) or n/available n (%). Missing-data percentages in Panel A use the total cohort of 771 patients as the denominator. Comparisons in Panel B used available observations and the Mann–Whitney U test or Fisher's exact test, as appropriate. Contrast volume was available in 94 excluded patients. Among the 97 patients excluded from the primary models, 91 lacked LVEF alone, two lacked diabetes status alone, three lacked contrast volume alone, and one lacked both LVEF and diabetes status. LVEF was available in only five excluded patients; therefore, a meaningful comparison of LVEF between groups was not possible. Multiple imputation was not performed. eGFR was calculated for all patients from admission serum creatinine, age, and sex using the 2021 CKD-EPI creatinine equation; therefore, no eGFR values were missing.

Abbreviations: ALT, alanine aminotransferase; APRI, aspartate aminotransferase-to-platelet ratio index; AST, aspartate aminotransferase; CA-AKI, contrast-associated acute kidney injury; CKD-EPI, Chronic Kidney Disease Epidemiology Collaboration; CRP, C-reactive protein; eGFR, estimated glomerular filtration rate; FIB-4, fibrosis-4 index; LVEF, left ventricular ejection fraction.

Supplementary Table S4. Calibration and incremental performance of FIB-4-based models.

A. Calibration

Model	n	Events, n	AUC	Hosmer–Lemeshow p	Brier score	Calibration slope
Primary FIB-4 model	674	83	0.684	0.141	0.100	0.90
Mehran score	766	93	0.646	0.459	0.101	1.04
Mehran score + FIB-4	766	93	0.666	0.437	0.100	1.00

B. Incremental value beyond the Mehran score and age

Model	n	Events, n	AUC	ΔAUC (95% CI)	DeLong p	LR test p
Mehran score + age	766	93	0.674	—	—	—
Mehran score + age + FIB-4	766	93	0.684	0.010 (−0.008 to 0.029)	0.282	0.025

The primary FIB-4 model included FIB-4, diabetes mellitus, hemoglobin, baseline serum creatinine, LVEF, and contrast volume. AUC values represent apparent performance in the study cohort. The Hosmer–Lemeshow test was based on deciles of predicted risk; fewer groups were formed when predicted risks were tied. Calibration slopes were estimated from 500 bootstrap resamples; values below 1 indicate optimism. In Panel B, FIB-4 remained associated with CA-AKI after adjustment for the Mehran score and age (OR 1.17, 95% CI 1.02–1.33; p = 0.021). n, number of patients; Events, CA-AKI events; LR, likelihood-ratio. Abbreviations: AUC, area under the receiver operating characteristic curve; CA-AKI, contrast-associated acute kidney injury; CI, confidence interval; LVEF, left ventricular ejection fraction; OR, odds ratio.
